# Clinical performance of non-invasive prenatal served as a first-tier screening test for trisomy 21, 18, 13 and sex chromosome aneuploidy in a pilot city in China

**DOI:** 10.1186/s40246-020-00268-2

**Published:** 2020-06-05

**Authors:** Yanhui Liu, Hailiang Liu, Yi He, Wanfang Xu, Qiulin Ma, Yuzhen He, Wei Lei, Guoquan Chen, Zheng He, Jiayi Huang, Jianan Liu, Yuanru Liu, Quanfei Huang, Fubing Yu

**Affiliations:** 1 Department of Prenatal Diagnosis Center, Dongguan Maternal and Child Health Hospital, Dongguan, 523112 Guangdong China; 2 CapitalBio Genomics Co., Ltd., Dongguan, 523808 China; 3 Dongguan Municipal Bureau of Health and Family Planning, Dongguan, 523112 Guangdong China; 4 Medical Department, Zunyi Medical College, Zunyi, China

**Keywords:** Non-invasive prenatal testing (NIPT), First-tier screening test, Positive predictive value (PPV), Sex chromosome aneuploidy, Advanced maternal age (AMA)

## Abstract

**Background:**

Cell-free fetal DNA (cffDNA) has opened up new approaches for non-invasive prenatal testing (NIPT), and it is often used as the second-tier test for high-risk pregnant women in detecting trisomy (T) 21, T18, and T13 after serum biochemistry screening. This study aims to discuss the clinical performance of NIPT as an alternative first-tier screening test for pregnant women in detecting T21, T18, T13, and sex chromosome aneuploidies (SCAs) in China.

**Methods:**

A total of 42,924 samples were recruited. The cell-free plasma DNA was directly sequenced. Each of the chromosome aneuploidies of PPV was analyzed. A total of 22 placental samples were acquired, including 14 FP and 8 TP samples. The placental verification of FP NIPT results was performed.

**Results:**

Among 42,924 samples, 281 (0.65%) positive cases, including 87 of T21, 31 of T18, 22 of T13, and 141 of SCAs were detected. For the detection of T21, the positive predictive value (PPV) was 78.46%, for trisomy 18, 62.96%, for trisomy 13, 10.00%, for SCAs, 47.22% in the total samples. For trisomy 21, the PPV was 86.67%, for trisomy 18, 80.00%, for trisomy 13, 20.00%, for SCAs, 56.52% in advanced maternal age (AMA) women. The PPV of T21 increased with age. For T18, the PPV showed an overall upward trend. For T13 and SCAs, PPV was raised first and then lowered. Placental verification of false positive (FP) NIPT results confirmed confined placental mosaicism(CPM) was the reason for false positives.

**Conclusions:**

This study represents the first time that NIPT has been used as a first-tier screening test for fetal aneuploidies in a pilot city with large clinical samples in China. We propose that NIPT could replace serum biochemistry screening as a first-tier test.

## Introduction

Non-invasive prenatal testing (NIPT) uses cell-free fetal DNA (cffDNA) in maternal plasma to detect certain genetic conditions during pregnancy. Recently, NIPT detects fetal chromosome abnormalities and has become increasingly common in prenatal care based on the clinical implementation of the new genomics-based technique [1], and this technique is very likely to replace standard prenatal trisomy (T) 21 screening for all pregnancies in the near future [2, 3]. Down syndrome (T21) is the most common cause of intellectual disability around the world, and it may affect approximately 1:500 pregnancies and is seen in 1:800 to 1:1000 live births [4].

NIPT is rapidly being adopted as to screening test for the detection of fetal aneuploidies of chromosomes 13, 18, and 21 [5, 6], and this method is also available for the detection of sex chromosome aneuploidies (SCAs )[7]. A recent meta-analysis showed that the weighted pooled detection rate (DR) and false-positive rate (FPR) for T21 were 99.7% (95% CI, 99.1–99.9%) and 0.04% (95% CI, 0.02–0.07%), respectively. The weighted pooled DR and FPR for T18 were 97.9% (95% CI, 94.9–99.1%) and 0.04% (95% CI, 0.03–0.07%), respectively, while the weighted pooled DR and FPR for T13 were 99.0% (95% CI, 65.8–100%) and 0.04% (95% CI, 0.02–0.07%), respectively [8] . The use of next-generation sequencing (NGS) technologies in NIPT has revolutionized the field, achieving sensitivities and specificities as high as 99% [9].

Since 2011, massively parallel screening (MPS) for fetal aneuploidies has become available in more than 60 countries [10]. NIPT has been rapidly integrated into prenatal care since the initial American College of Medical Genetics and Genomics (ACMG) statement in 2013 [11]. Therefore, several professional societies have recommended that NIPT be offered to pregnant women at high risk for having a fetus with autosomal aneuploidy. These statements are summarized in Table 1 [11–16]. In addition, the American College of Obstetricians and Gynecologists (ACOG) recommended that prenatal screening for aneuploidy should be offered to all women rather than only women of advanced maternal age (AMA) [17].

In many countries, genomics-based NIPT is now adopted or being implemented in public laboratories as a second-tier prenatal screening test for autosomal aneuploidies. However, there are limited data on the impact of implementing cfDNA as a first-tier screening test on ongoing pregnancy management and outcome and as such, the optimal aneuploidy screening model for a general pregnant population has yet to be determined. NIPT has been available in China since 2011, it is currently recommended as a second-tier test after serum biochemistry screening, too. In the USA and Netherlands, the attitudes of health professionals toward offering NIPT as a first-tier screening test are generally favorable [13, 18]. In this study, we chose a pilot city to perform NIPT as a first-tier screening test, and our aim was to promote NIPT for aneuploidy screening as a first-tier screening test for the general population in China.

**Table 1:** Statements on non-invasive prenatal screening for fetal aneuploidy

**Institution**	**Statement**
American College of Medical Genetics and Genomics(ACMG)	NIPT is a screening test to identify pregnancies at risk for common autosomal aneuploidies (e.g., trisomy 21, 18, and 13). Some laboratories also offer screening for sex chromosome aneuploidie s[11, 12]. NIPT for fetal aneuploidy has arrived.
National Society of Genetic Counselors (NSGC)	NIPT as an option for aneuploidy assessment in pregnancy: Peer-reviewed data currently supports NIPT as a screening tool for select population s[13].
Israeli Society of Medical Geneticists (ISMG)	It may be advantageous to integrate NIPT with the current screening modalities as part of the screening program for fetal aneuploidy in Israe l[14, 15].
The International Society of Ultrasound in Obstetrics and Gynecology (ISUOG)	NIPT as a first-line screening test. Using NIPT on intermediate- or low-risk patients might be endorsed as a widely available option only when new data emerge and NIPT costs decreas e[16].

## Materials and methods

### Patients

This study was a retrospective cohort study conducted from January 2016 until February 2017. After pretest counseling, written consent was obtained. Maternal blood samples were collected from women 6–32 weeks of pregnant age. Our main objective in this study was to detect T21, T18, T13, and SCAs. Inclusion criterion was (1) local household registration pregnant woman. Exclusion criteria were (1) pregnant women with chromosomal abnormalities, (2) pregnant women who have received stem cell therapy, transplant surgery, (3) received allogeneic blood products within 1 year, and (4) received immunotherapy within 4 weeks.

### Samples prepare and sequencing

When NIPT was performed, maternal blood samples were obtained from the participants by collecting 5 to 10 mL of peripheral blood into tubes primed with EDTA. Within 8 h, the samples were prepared for cfDNA sequencing as previously described [19]. The blood samples were first centrifuged at 1600×*g* for 10 min at 4 °C to separate the plasma from the peripheral blood cells and then carefully transferred to a polypropylene tube and centrifuged at 16,000×*g* for 10 min at 4 °C to pellet the remaining cells. The cfDNA extraction, library construction, quality control, and pooling were performed according to the instructions of the JingXin Fetal Chromosome Aneuploidy (T21, T18, T13) Testing Kits (CFDA registration permit No. 0153400300). For DNA sequencing, we pooled 15~20 libraries and sequenced ~200 bp reads on a JingXin BioelectronSeq 4000 System (CFDA registration permit No. 20153400309 )[20]. To identify the fetal autosomal aneuploidies T21, T18, and T13, we used the combined GC-correction and Z score testing methods described in our previous paper [19]. The copy number variations (CNVs) of the fetal and maternal chromosomes were classified using the modified Stouffer’s Z score method that we described in another paper [21]. All reports were double-checked. Positive NIPT results were recommended for invasive diagnosis. Negative NIPT results were monitored during routine antenatal care.

Fetal DNA concentration was calculated by Y chromosome (FC%) according to the instructions of the JingXin Fetal Chromosome Aneuploidy (T21, T18, T13) Testing Kits. Fetal DNA concentration was calculated by FC%(Y)=(R−Rr)/2Rr (1), which represents the algorithm for total FC%. FC%(T) was calculated by the proportion of triploidy: *Z* = (R−Rr)/SD (2). Thus, FC% = 2*CV*Z, according to (1) and (2). CV is the coefficient of variation. Sex chromosome aneuploidy detection using our previously described method [19]. Simply, first, Z scores for the X and Y chromosomes were generated as described for the autosomes. Then, a least-squares method was applied to establish the relationship between the X and Y chromosomes of a female fetus based on the formula Z_x_ = r × Z_Y_ + b represents the Z score for the X chromosome and *r* represents the coefficient between the X chromosome and Y chromosome. See Liao’s paper [19] for details. A cutoff value of Z score > 3 was used to determine whether the ratio of chromosomes was increased and also the fetal trisomy 21, 18, and 13 was present, as described previously [19, 21]. Here, each chromosome with an absolute value of the Z score greater than 3 was marked with chromosome aneuploidies or CNVs.

## Results

### Study population

From January 2016 until February 2017, 42,924 maternal blood samples were collected. Table 2 summarizes the demographic characteristics. The median maternal age was 30.3 (range 18–47) years, and the median gestational age was 15.5 (range 8–32) weeks. Most samples were collected from participants with maternal ages below 35 years (78.7%). In China, NIPT is recommended after serum biochemistry screening in the second trimester. Thus, the vast majority of samples were collected in the second trimester (36,173, 84.3%).

**Table 2:** Demographic characteristics of pregnant women undergoing NIPT

**Characteristic**	**Total (*n* = 42,924)**
GA at NIPT (weeks)	
First trimester(6–13 weeks)	6471
Second trimester(14–27 weeks)	36,173
Third trimester(≥ 28 weeks)	218
Unknown	62
Maternal age (years)	
< 30 years	20,137
30–34 years	13,647
35-39 years	7991
≥ 40 years	1144
Unknown	5
Pregnancy	
Singleton pregnancy	42,257
Twin pregnancy	667
Advance maternal age (>35 years)	9135

*GA* gestational age

**Table 3:** NIPT results in total samples and AMA women samples

NIPT	Total samples					AMA women				
	T21	T18	T13	SCAs	Total	T21	T18	T13	SCA	Total
Total positive cases	87	31	22	141	281	40	13	7	29	89
Frequency	1/493	1/1385	1/1951	1/304	1/153	1/228	1/703	1/1305	1/315	1/103
TP	51	17	2	51	121	26	4	1	13	44
FP	14	10	18	57	99	4	1	4	10	19
PPV`	78.46%	62.96%	10.00%	47.22%	55.00%	86.67%	80.00%	20.00%	56.52%	69.84%

*TP* true positive, *FP* false-positive, *PPV* positive predictive value, *T21* trisomy 21, *T18* trisomy 18; *T13* trisomy 13; *SCAs* sex chromosomal aneuploidies, *AMA* advanced maternal age

**Table 4:** Three duplicate biopsy samples of fetal side and maternal side

Case	NIPT Result	Maternal side			Fetal side		
		Center point	Middle point	Edge point	Center point	Middle point	Edge point
1	T7	47,XX,+7[15]/46,XX[85]	Normal	Normal	Normal	Normal	47,XX,+7[15]/46,XX[85]
2	T7	47,XX,+7[40]/46,XX[60]	/	/	Normal	/	/
3	T7 and T2	48,XX,+2[10],+7[10]/46,XX[80]	/	/	48,XX,+2[10],+7[10]/46,XX[80]	Normal	Normal
4	T7	47,XY,+7[10]/46,XY[90]	Normal	47,XY,+7[70]/46,XY[30]	Normal	Normal	Normal
5	T7	47,XX,+7[65]/46,XX[35]	47,XX,+7[65]/46,XX[35]	47,XX,+7[65]/46,XX[35]	47,XX,+7[5],46,XX[95]	47,XX,+7[5],46,XX[95]	47,XX,+7[5],46,XX[95]
6	T7	47,XX,+7[20]/46,XX[80]	47,XX,+7[20]/46,XX[80]	47,XX,+7[20]/46,XX[80]	Normal	Normal	Normal
7	T7	Normal	Normal	Normal	47,XX,+7[20]/46,XX[80]	Normal	Normal
8	T7	Normal	Normal	Normal	47,XX,+7[15]/46,XX[85]	47,XX,+7[10]/46,XX[90]	Normal
9	T7	Normal	Normal	Normal	47,XX,+7[5]/46,XX[95]	Normal	47,XX,+7[15]/46,XX[85]
10	T7	47,XX,+7[80]/46,XX[20]	47,XX,+7[80]/46,XX[20]	47,XX,+7[70]/46,XX[30]	47,XX,+7[60]/46,XX[40]	47,XX,+7[30]/46,XX[70]	47,XX,+7[50]/46,XX[50]
11	T7	47,XX,+7[75]/45,XO[20]/46,XX[5]	46,XO,+7[65]/46,XN[35]	47,XX,+7[75]/46,XX[25]	47,XX,+7[75]/46,XX[25]	47,XX,+7[65]/45,XO[20]/46,XN[15]	47,XX,+7[60]/46,XX[40]
12	T7	47,XX,+7[70]/46,XX[30]	47,XX,+7[80]/46,XX[20]	47,XX,+7[70]/46,XX[30]	47,XX,+7[50]/46,XX[50]	47,XX,+7[60]/46,XX[40]	Normal
13	T7	47,XX,+7[25]/46,XX[75]	47,XX,+7[10]/46,XX[90]	47,XX,+7[15]/46,XX[85]	47,XX,+7	47,XX,+7[25]/46,XX[75]	47,XX,+7[10]/46,XX[90]
14	T7	Normal	Normal	Normal	Normal	Normal	Normal

/: no sample

**Table 5:** Details of true-positive NIPT samples with the validation results

**Case**	**NIPT result**	**Umbilical cord tissue**	**Fetal tissue**	**Placental tissue**	
				**Fetal side**	**Maternal side**
Case 15	T21	T21	/	/	/
Case 16	T21	/	T21	47,XN,+21[75]/46,X N[27]	T21
Case 17	T21	T21	/	NA	NA
Case 18	T21	T21	/	T21	T21
Case 19	T18	T18	/	T18	T18
Case 20	T21	/	/	T21	T21
Case 21	T21	T21	/	T21	T21
Case 22	T21	/	/	T21	T21

/ No sample

### NIPT results for T21, T18, T13, and SCAs in total samples

**Fig. 1:**
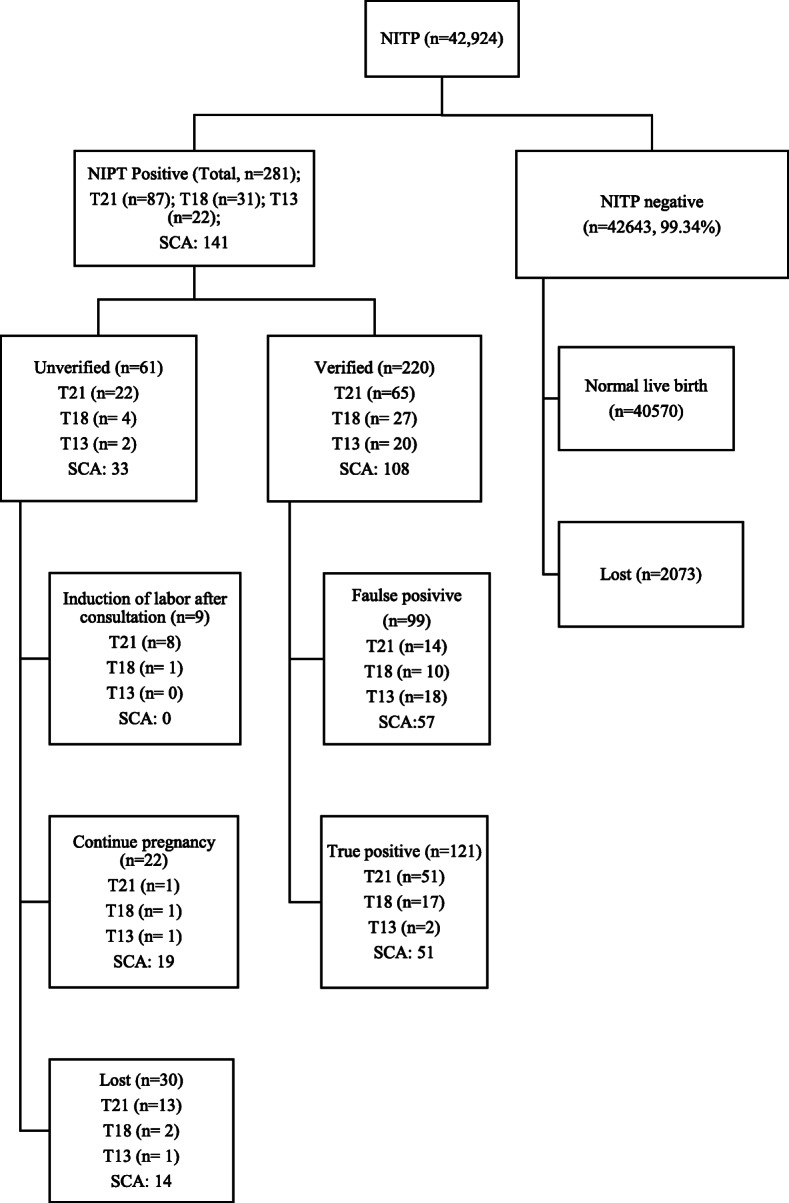
Flowchart of non-invasive prenatal test (NIPT) results and clinical outcome of pregnant women T21: trisomy 21; T18: trisomy 18; T13: trisomy 13; SCA: sex chromosomal aneuploidies

The flowchart is shown in Fig. 1. A total of 42,924 samples were recruited including 281 (0.65%) positive cases. Of these 281 cases, there were 87 of T21 (1/493), 31 of T18 (1/1385), 22 of T13 (1/1951), and 141 (1/304) of SCAs. Prenatal diagnostic testing results were obtained to verify the abnormal results of the NIPT predictions. Of these 281 cases, there were 220 (78.29%) cases underwent additional prenatal diagnostic testing, which confirmed 51 cases of T21, 17 of T18, 2 of T13, and 51 of SCAs. Moreover, the positive predictive value (PPV) for each test was assessed. For trisomy 21, the PPV was 78.46%, for trisomy 18, 62.96%, for trisomy 13, 10.00%, for SCAs, 47.22% (Table 3).

### NIPT results for T21, T18, T13, and SCAs in AMA women

In this study, there were 9135 women of advanced maternal age (≥ 35 years), accounting for a relatively large proportion. NIPT predicted 40 (1/228) of T21, 13 (1/703) of T18, 7 (1/1305) of T13, and 29 (1/315) of SCAs in AMA women. For trisomy 21, the PPV was 86.67%, for trisomy 18, 80.00%, for trisomy 13, 20.00%, for SCAs, 56.52% in AMA women. The frequency of T21 and T18 in AMA women was much higher than the total samples. Simultaneously, the PPV of T21 and T18 in AMA has risen a lot compared with the total samples (Table 3).

### The relationship between different PPV and age

**Fig. 2:**
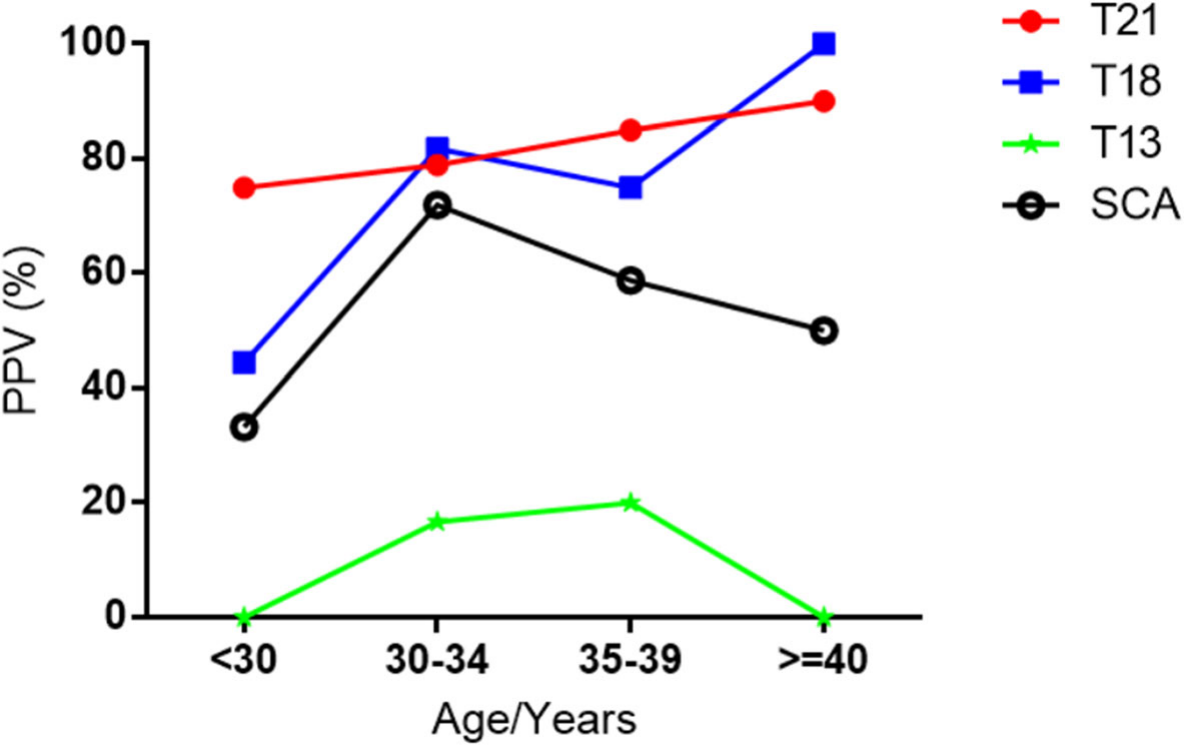
The relation between positive rate and pregnant age for trisomy 21, trisomy 18, and trisomy 13 T21: trisomy 21; T18: trisomy 18; T13: trisomy 13; SCA: sex chromosomal aneuploidies

We also assessed the relationship between different PPV and age. People were categorized into three groups according to age: > 30 years, 30–34 years, 35–39 years and > 40 years. PPV of T21 increased with age. For T18, the PPV showed an overall upward trend. For T13 and SCAs, PPV was raised first and then lowered. The PPV for T13 was the highest in the 30–34 years group, and for SCAs, 30–34 years group was the highest (Fig. 2).

### Fetal cfDNA concentrations

Many studies have found that the concentration of fetal cfDNA in maternal plasma can influence the accuracy of NIPT [22, 23]. Of the 42,924 pregnancies, 0.54% of the samples required a second blood draw because of low fetal cfDNA concentration, thus all pregnancies estimated to have fetal cfDNA concentrations > 4%.

**Fig. 3:**
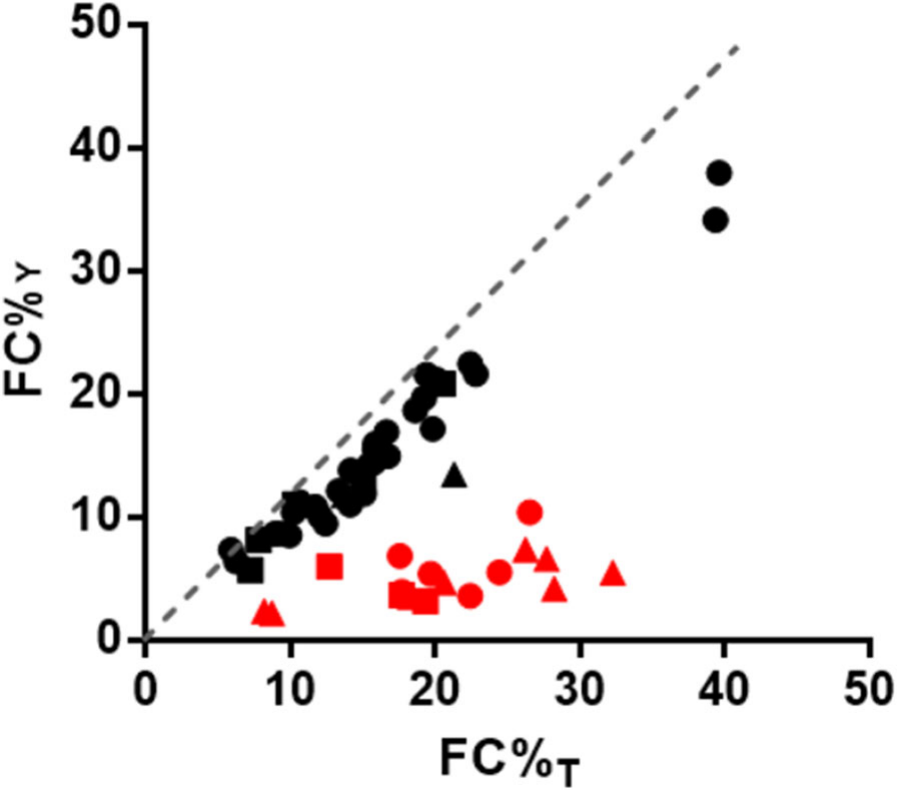
The relationship between FC%_Y_ and FC%_T_ between true-positive and false-positive samples. True-positive samples are shown as black spots, false-positive samples as red circles. T21 cases: dot; T18 cases: quadrate; T13 cases: triangle FC%: Fetal DNA concentration, FC%_Y_: Fetal DNA concentration calculated by Y chromosome, FC%_T_: Fetal DNA concentration calculated by the proportion of triploid

There were 54 pregnancies of male fetuses had underwent additional prenatal. Of these 54 cases, there were 37 TP (including 30 of T21, 5 of T18, and 2 of T13) and 17 FP (including 6 of T21, 3 of T18 and 8 ofT13 predicted by NIPT). We applied two methods to calculate fetal concentration in male fetuses. Fetal DNA concentration calculated by the Y chromosome (FC%Y), and fetal DNA concentration (FC%T) calculated by the proportion of triploidy. Importantly, there existed a certain relationship between the fetal concentrations calculated by the two methods between TP and FP results. When we plotted these data, the TP cases were near the correlation coefficient. While all the FP cases were far away from that line, in other words, they were closer to the horizon. (Fig. 3).

### NIPT results for CNVs

Besides, we have also analyzed the CNVs, because this technology is genome-wide sequencing. There were 56 cases of CNVs. One case had developmental malformations shown by ultrasound, and 38 cases had undergone further diagnosis with amniocentesis, confirmed 11 true-positive and 27 false-positive results. The PPV of CNVs was 28.95%. Of these 11 true positive cases, a pregnant woman insisted on continuing her pregnancy, and she gave birth to a baby with 5p deletion syndrome.

### Placental verification of Trisomy 7(T7) FP NIPT results

Besides, we have also analyzed other chromosome aneuploidies, and all patients with a positive NIPT result were recommended for invasive prenatal testing. Invasive prenatal testing confirmed some of the discordant results of other chromosome aneuploidies. We found all the T7 predicted by NIPT were discordance with the invasive prenatal testing results. Thus, we want to research the reasons for the discordance by verification experiment on the postpartum placenta. After pretest counseling, some patients were willing to donate the placenta for further research. From October 2016 until October 2017, a total of 22 placental samples were acquired, including 14 FP (all the 14 cases were T7 predicted by NIPT) and 8 TP (including 7 of T21 and 1 of T18 predicted by NIPT) samples.

Six placental biopsies in triplicates, three from the maternal side and three from the fetal side, were obtained. The triplicate samples of the maternal side and fetal side were from the center, middle, and edge of the placenta. Each case showed varying degrees of confined placental mosaicism (CPM) except case 14 by NGS (Table 4). The chimeric ratio of the three samples in the maternal side of case 5 is consistent, so as was the fetal side. In case 6, only the maternal side showed CPM, while the fetal side was normal. Cases 7, 8, and 9 displayed CPM on the maternal side. Moreover, the other samples showed different placental chimerisms in different positions.

There were 8 cases of TP NIPT samples (Table 5). These pregnancies were interrupted after confirmation. The validation results of the umbilical cord tissue and fetal tissue were concordant with the NIPT results, and case 16 showed a level of 75% for T21 at the fetal side.

## Discussion

### Efficiency of NIPT in our study

NIPT has been widely used to detect T21, T18, and T13 for a number of years [28], but due to lacking large-scale clinical studies on its efficacy in the general population, it has not yet become a first-tier test in China. To provide a large clinical dataset supporting NIPT as a first-tier test, we chose Dongguan, China, as a pilot city. Over the past year, we had recruited 42,924 pregnant women. Thus, we hope to promote NIPT as a first-tier screening test for all pregnant women in China.

We used positive predictive value (PPV) to evaluate NIPT in this study. The PPV for T21 was 78.46%, and for T18, T13, SCA was 62.96%, 10.00%, 47.22%, respectively. In several recent studies, the PPV range of T21 was 65–94%, T18 was 47–85%, and T13 was 12–62% [27–29]. Our results fall within this range except for the PPV of T13. The PPV of T13 in this study was slightly lower than the above literature. The reason is that our NIPT was used as a first-tier test, and no entry criterion was established to exclude situations, which might decrease the PPV. Furthermore, this NIPT screening was free supported by the government, eligible pregnant women were more willing to participate. Therefore, patients had not previously undergone serum biochemistry screening.

The frequency of T21 was 1/493, which was nearly 2 times higher than the proportion (1/800–1/1000) of newborn infants born with Down syndrome. The reason is that, on the one hand, miscarriages; on the other hand, half of the actual pregnant women will terminate this pregnancy [30]. We had also detected 141 cases of SCAs, and the frequencies of SCA were consistent (1/304, 1/315) between total samples and AMA women. Different pregnancies characteristics show different PPV, and the PPV for SCAs in AMA women(56.52%) was slightly higher than in the total samples (47.22%). Similarly, the PPV of T21/T18/T13 in AMA was all higher than the total samples in the present study. In a previous study, the efficiency of NIPT among 3585 advanced maternal age women was higher than the total samples too [7].

NIPT for fetal aneuploidy has rapidly transformed the global prenatal screening landscape. But, few countries use it as a first-tier screening application. A previous study [31] of NIPT as a first-tier screening test in the Netherlands showed a high efficiency, and this study was granted by a governmental license to evaluate the implementation. It showed the PPV of 96% for trisomy 21, 98% for trisomy 18, and 53% for trisomy 13, which was higher than this present study. Our present study was a retrospective study in a pilot city. Thus, NIPT used as a first-tier screening test will be better implemented if there is a governmental license granted.

Recently, more relaxed guidelines have been suggested screening for CNVs can be performed routinely for younger women because microdeletions are more frequent than aneuploidies in this situation [32]. Previous studies reported a variable performance of NIPT for the detection of specific CNVs, with only low to moderate PPVs. The PPV for CNVs was 28.95% in the present study, which was similar to Chen’s paper [24].

In recent years, many studies have also reported NIPT screened for other chromosome aneuploidy [33]. But, the PPVs for other chromosome aneuploidy were very low. On the one hand, these aneuploidies are less prevalent. On the other hand, many of them have high confined placental mosaicism (CPM). NIPT used cell-free fetal DNA to sequence, and the primary source of cell-fetal DNA is apoptosis of placental cells from the cytotrophoblast [34], which is not always representative of the fetus. There is a situation that a chromosomal abnormality occurs only in the placenta but not in the fetus, which is known as CPM. Multicenter studies have reported that the frequency of CPM was around 1–2% [35]. In CPM cases, the cytogenetic abnormality, most often trisomy, is confined to the placenta [36]. Thus, the cfDNA in maternal circulation and the actual fetal karyotype would be discordant. Thus, we found that the main reason for T7 FPs was CPM, at the same time, CPM showed significant regional variation. But, FPs due to CPM was not unique, and chimerism did not show a tendency toward the position. Thus, NIPT is a screening test. For pre-counseling for NIPT, women who choose should be well informed about the accuracy, reliability, false-positive and false-negative rates. So, NIPT screened for other chromosome aneuploidy needs more validation to determine accurately its detection rate, and false-positive rate.

Before NIPT as a first-tier screening, pregnant women need to undergo biochemical serum. Serum screening test has a higher false-positive rate than NIPT. Thus, a high percentage of women were required to undergo further diagnostic testing, along with the attendant procedure-related miscarriage s[3]. However, when using NIPT, only a small number of pregnancies were defined as “high risk”. That is, NIPT significantly reduced the potential need for invasive diagnostic testing. Furthermore, conventional screening for aneuploidies does not include SCAs screening, while ACMG recommends informing all pregnant women that NIPT may be expanded to screen for SC A[25]. NIPT displays the hallmarks of a screening method suitable for T21, T18, T13, and SCAs in this study.

### Comparison between NIPT and serum screening

Prenatal screening for trisomies based on the analysis of biochemical markers in maternal serum has become available in many countries. Among these biochemical markers in maternal serum, free b-human chorionic gonadotrophin (free b-hCG) and pregnancy-associated plasma protein-A (PAPP-A) are the most widely used and most valuable biochemical markers.

Maternal serum-free b-human chorionic gonadotrophin (free b-hCG) and pregnancy-associated plasma protein-A (PAPP-A) has been shown to be of value in the biochemical markers that have been investigated. The combined test uses these markers in conjunction with nuchal translucency measurements and is estimated to achieve a DS detection rate of 80% to 85% at a 5% false-positive rate [26]. Before this project, serum screening was the first-tier screening test for T21 in China. We will perform Down’s screening in two stages in China. The first-trimester screening offers a noninvasive option for the early detection of aneuploidy pregnancies. To calculate the risk of fetal suffering from Down syndrome, this screening is done by a combination of two biochemical markers (1) serum-free b-human chorionic gonadotrophin (free b-hCG) and (2) pregnancy-associated plasma protein-A (PAPP-A )[37], which is also called combined first-trimester screening (CFTS). In the second trimester of pregnancy matemal serum α-fetoprotein (AFP) levels are, on average, lower in pregnancies associated with fetal Down syndrome than in unaffected pregnancies, and some centers offer antenatal screening for Down syndrome based on serum AFP as well as maternal ag e[26]. If serum screening is a high risk, the pregnancy will undergo interventional puncture, such as chorionic villus sampling and amniocentesis.

From 2013 to 2016, our center screened 198,079 serological samples for T21, resulting in a DR of 70% and a PPV of almost 10%. If all 42,924 of the samples in this study had been detected by serum screening, 36 of the T21 fetuses would have been found (51*0.70). Thus, 15 of the T21 fetuses would have been missed. However, serum screening has a higher FP rate than that of NIPT, which would have led to more amniocentesis. The PPV of serum screening in our center was 10%, so 459 FP results would have occurred in 42,924 samples. These patients would have undergone PD by amniocentesis. In the literature, the procedure-related fetal loss rate is approximately 1.0 %[1] for amniocentesis. Thus, approximately 5 (459*1%) pregnancies would likely have been lost after amniocentesis. However, in our NIPT program, only 14 FP results of T21 were found. At the expected fetal loss rate, NIPT would not cause fetal loss. Therefore, NIPT has an absolute accuracy advantage over serum screening.

The market price of NIPT has already reduced to about 1500RMB (US$214), and the serum screening is about 120 RMB (US$17) in China in 2019. Further fall in NIPT cost is expected for good reasons. As the cost decreases, NIPT will become more and more popular. A study showed that the majority (72%) of Dutch obstetric health professionals are in favor of replacing serum screening by NIPT [38]. Similarly, several studies proposed that it could replace serum biochemistry screening to act as a first-tier screening test [39]. Besides, NIPT technology is very mature to perceive as a routine prenatal test, and ACMG recommends NIPT to replace conventional trisomy screening techniques in pregnant women of different ages.

## Conclusions

This study represents the first time that NIPT has been used as a first-tier screening test for fetal aneuploidies in a pilot city in a large sample population. The data have potential significance in demonstrating the usefulness of NIPT profiling for T21, T18, T13, and SCAs. Moreover, NIPT screened for other chromosome aneuploidy needs more validation to determine accurately its detection rate and false-positive rate. At last, we propose that NIPT could replace serum screening as a routine practice.

## Data Availability

The datasets used and/or analyzed during the current study are available from the corresponding author on reasonable request.

## Abbreviations

NIPT: Non-invasive prenatal testing
MPS: Massively parallel sequencing
PPV: Positive predictive value
cfDNA: Cell-free DNA
NGS: Next-generation sequencing
Chr: Chromosome
TP: True positive
FP: False positive
FN: false negative
GA: Gestational age
SCA: Sex chromosome aneuploidy
DR: Detection rate
FPR: False-positive rates
AMA: Advanced maternal age
CF%: Fetal DNA concentration
